# Supplementary material for: Identifying a common backbone of interactions underlying food webs from different ecosystems
Source: Nat Commun. 2018 Jul 4;9:2603. doi: 10.1038/s41467-018-05056-0 (PMC6031633; doi:10.1038/s41467-018-05056-0)
Supplement: Supplementary file 1 — Supplementary Information [file 41467_2018_5056_MOESM1_ESM.pdf]

# Identifying a common backbone of interactions underlying food webs from different ecosystems

Bramon Mora et. al.

# Supplementary Note 1

## Structural differences across ecosystems

Using the estimated alignments between food webs, we calculated four dissimilarity matrices $\hat{E}$ containing the pairwise distances between the 411 food webs based on each of the four alignment quality measures defined in the Supplementary Methods section. These dissimilarity matrices allow us to test whether or not food webs from different ecosystems generally share a common structure. To test this, we used a permutational multivariate analysis of variance (PERMANOVA; 1). First, we tested for general differences in the alignment centroids among the different ecosystem types when using the whole dissimilarity matrix (Supplementary Table 1). To avoid aligning networks with very different sizes, we then repeated the test using only a subset of the networks. In particular, we repeated the test considering the biggest possible subset of networks from our dataset, such that the biggest network was at most twice as big as the smallest network (Supplementary Table 1). Finally, we performed a Principal Coordinates Analysis (PCoA) of the whole dissimilarity matrix to visualize the observed differences (Fig. 2 of the main text).

## Pairwise comparisons between ecosystems

In this section, we first individually studied the differences between every pair of ecosystem types. Specifically, we compared the 34 estuaries, 87 lakes, 148 marine ecosystems, 88 streams and 54 terrestrial ecosystems using the alignment measure $A$ (Supplementary Table 2). Then, we performed a principal coordinate analysis of every comparison (Supplementary Fig. 1).

## Connectance and path likelihood

One of the necessary conditions for the existence of a backbone of interactions is that the best aligned species need to form a connected component. The two measures that we used to test this idea are the connectance and path likelihood. For every network, we found that the substructures formed by the sets of best aligned species tend to present both a high

connectance and path probability when compared to equal sized random subsets of species (Supplementary Fig. 3).

## Links removals: alternative measure for identifying the backbones of interactions

To compare the backbones of interactions found for every network, we used a different measure of network similarity as an alternative to backbone alignment. Because the backbones are defined by determining the most-overlapped interactions across alignments, it was important that the measure of network similarity not consider differences in the number of species comprising the backbone but only differences in the interactions forming it. In particular, we defined a measure based on "link removals", where the distance between two backbones $C$ and $D$ is calculated as the minimum set of interactions that need to be removed from $C$ and $D$ before they turn into isomorphic structures. To estimate such a distance for every pair of backbones, given two structures made of $n$ links, we recursively try all the potential $k$-link-removal permutations on both networks until we find isomorphic structures. That is, for one link removal, for example, we would test whether or not there is any combination of one-link removals that produces two isomorphic structures made of $n-1$ links.

For this alternative analysis, we concentrated on the backbones of interactions made up of the six most-overlapped links, since this method is computationally very expensive for large backbones and this is the number of links of the smallest network in our dataset. Comparing the backbones using the measure of link removals, we looked for the two structures that could explain most of the observed backbones (Fig. 7). In particular, we found that these two structures together can explain approximately 60% of the networks' backbones within one 'link removal'. Importantly, the structures found are consistent with the previous results, since they are substructures of the backbones shown in Fig. 4 of the main text.

## Many-link backbones of interactions

Given the clustering analysis performed for a particular backbone size $k$, we can assess how distinct the observed clusters are by analyzing the 95% confidence ellipses (see Fig. 4 of

the main text). In particular, we looked at the amount of overlap between these ellipses and found that it drastically increased when the backbone size was $k > 15$ (Fig. 8). This could highlight the fact that the noise associated with the shape of the backbones increases with their size, which is not surprising because the periphery around the backbones seem to differ across networks (Fig. 3 of the main text).

### Backbones of interactions for each ecosystem

Comparing the backbones of interactions that one would independently find for every ecosystem is nuanced for two main reasons. First, although the existence of backbones of interactions underlying all food webs can be assessed by looking at the alignment transitivity and connectance, we estimated the shapes of such backbones by overlapping the different networks in question. This overlap provided us with an intuitive shape of what the backbones might look like. However, this shape can entail a lot of variability, and one ideally wants to include as many networks as possible to obtain results that are robust to this variability. Focusing on individual ecosystems necessarily limits the number of networks we can use in the analyses, and, therefore, reduces the strength of our results. Second, we do not have the same number of networks for every ecosystem, which also makes the comparison of the resultant backbones in some way uncertain.

Despite these subtle but important distinctions, we repeated the analysis described in the main text for each ecosystem individually. That is, for every ecosystem, we identified every network's backbone of interactions made up of the $k$ most-overlapped links. In this case, we focused only on the backbones made of 6 links, which is the size of the smallest network in our database. For every ecosystem type $t$, we then aligned the corresponding backbones and generated the dissimilarity matrices $E_6^t$, where every element $e_{ij|6}$ in these matrices is the optimal alignment cost between the 6-link backbones from any network $i$ and $j$ (Eq. 3 of the main text). Similar to what we found across all ecosystem types, the analysis of these dissimilarity matrices using clustering techniques revealed the existence of two backbones of interactions for any dissimilarity matrix $E_6^t$ (Fig. 9). This implies that two distinct backbones appear regardless of the ecosystem used for the analysis, which could indicate that the backbones found for the entire dataset are general across ecosystems.

To test this idea, we also studied whether or not the clustering found for the entire dataset agrees with the clustering found for the different ecosystems. Using a PERMANOVA analysis, we compared the dissimilarity matrices $E_6^t$ to the results for the clustering analysis of the entire dataset. With the exception of estuary food webs ($p = 0.951$), we found this clustering to be a significant predictor of every dissimilarity matrix $E_6^t$ ($p < 0.01$). This implies that the clustering found for the entire database agrees with the clustering found for the individual ecosystems. The inconclusive results found for the estuarine food webs may relate to an ecological phenomenon or simply be the result of this ecosystem exhibiting the smallest sample size with only 34 networks.

## Trophic level of the backbones

For every network, we classified the trophic level $h$ of the species based on whether or not they were basal ($h = 0$), intermediate ($h = 1$) or top species ($h = 2$). Then, for each six-link backbone of interactions, we averaged the trophic level of the species across all networks. We found no significant differences in the trophic levels of the species forming each backbone, with one backbone showing a $h = 0.97 \pm 0.20$ and the other backbone showing a $h = 1.01 \pm 0.22$.

## Compartmentalized structure of food webs

To measure the degree of compartmentalization across the food webs, we used the quality measure of modularity (2). To calculate this, we used the algorithm presented by Leicht, E. A. and Newman M. E. J. (3), which estimates both the measure of modularity and the optimal number of modules for any given network. We found that almost every network of our dataset showed some degree of compartmentalization, since the networks in our dataset have, on average, $3.45 \pm 1.18$ modules. In particular, these networks show an average modularity of $0.25 \pm 0.10$, with only two networks showing null modularity.

# Supplementary Methods

## Alignment algorithm

Given two food webs, we presented an alignment algorithm that aims to find the best matching of species in one network to species in the other. Starting from a random alignment between these food webs, we use a stochastic optimization algorithm to progressively modify this initial alignment and minimize an alignment cost function that increases when species playing similar ecological roles are paired. During the course of our study, we identified three crucial aspects regarding the alignment cost function that one needs to consider: (1) a pairwise measure of role similarity between species, (2) the degree of the alignment, and (3) the contribution of unpaired species.

*Pairwise similarity between species roles*

As noted in the main text, we use the definition based on the idea of network motifs (4)—the unique $n$-species subnetworks describing all patterns of interactions between $n$ species—to measure the structural role of species in our dataset. For example, considering two-species food-web motifs would imply studying the distinct subnetworks $a \leftarrow b$ and $c \leftrightarrow d$. The first motif $a \leftarrow b$ represents species $a$ consuming species $b$ and defines two unique positions: the consumer and resource. On the other hand, the second motif $c \leftrightarrow d$ describes two different species consuming each other and represents a single unique position, since $c$ and $d$ are indistinguishable. Following this scheme, species of any given food web could be characterized by a "motif-role profile" accounting for the number of times that they appear in each of the three unique positions of the two-species motifs. The resolution of this motif-role profile can be extended to also consider three-species food-web motifs, which define 13 additional patterns of interaction and 30 new unique positions. As a result, the motif-role profile of any species can be defined as $\vec{c}_a = \{c^2_{a1}, c^2_{a2}, c^2_{a3}, c^3_{a1}, \ldots, c^3_{a30}\}$, where all $c^2_{aj}$ and $c^3_{ak}$ define the frequencies that any species $a$ appears in the two-species-motif positions $j$ and the three-species-motif positions $k$, respectively. The pairwise role similarity between any two species can then be calculated using those motif-role profiles and by means of Eq. (1) in the Methods section of the main text. Other motifs sizes could

also be considered if desired; however, there are 199 four-species motifs and—given the alignment strategy described below—such additional information would likely be redundant and computationally inefficient.

### *Degree of the alignment*

Following the definition of the pairwise role similarity, we can now compare the roles of individual species from any two food webs $A$ and $B$; given an alignment between those networks, we can also compute the overall similarity between them. In the paper, we described two possible strategies for aligning networks: pairing species to species and pairing species' neighbors to species' neighbors. The former strategy is characterized by optimizing Eq. (2) in the Methods section of the main text. Notably, this cost function would focus on minimizing the sum across every species-species pairings and might ignore the actual structure of food webs (Supplementary Fig. 10). Instead, the latter strategy is characterized by optimizing Eq. (3) in the Methods section of the main text and pairs up species based on the motif-role profile of their neighbors (Supplementary Fig. 10).

The advantage of pairing species' neighbors to species' neighbors over pairing species to species can be understood using the example presented in Supplementary Fig. 10. In this example, we have two simple food webs aligned as $\{(A,a),(B,b),(C,c),(D,d),(\emptyset,e)\}$, where every element $(i,j)$ represents the pairing between any given two species $i$ and $j$ from each of the networks. Although this alignment is optimal according to any of the two strategies presented here, there are other possible alignments that are as good. In particular, pairing species to species we would find 4 indistinguishable alignments (Supplementary Fig. 11). The reason is that the cost function associated to such strategy will reach a minimum as long as the individual species' roles match. Therefore, the pairing $(A,a)$ will be as probable as the pairing $(A,e)$ and independent from the pairing of the neighbors of $A$. In contrast, pairing species' neighbors to species' neighbors only implies finding 2 indistinguishable results because the cost of pairing $(A,a)$ will depend on the pairing of the neighbors of $A$ (Supplementary Fig. 11).

*Contribution of unpaired species*

The first aspect to consider regarding the contribution of unpaired species is the penalty $\xi_x$ used in Eq. (3). This penalty could be defined as simply the number of neighbors that have not been paired to any species. However, we are interested in allowing species to remain unpaired if we cannot find them a proper match, and this approach would over-penalize non-pairing. Instead, we define this penalty for the alignment $x = (a, b)$ based on the unpaired neighbors of the species with a higher degree (i.e. the non-paired neighbors of species $a$ when $n_a \geq n_b$ and the non-paired neighbors of $b$, otherwise). Following this, we can rewrite the term $\xi_x$ as $\xi_{(a,b)} = \max\left(k_{x_\alpha}, k_{x_\beta}\right) \times (1 - \varepsilon)$, where $k_{x_\alpha}$ (and $k_{x_\beta}$) is the number of neighbors of $a$ (and $b$) that are not paired with a neighbor of $a$ (and $b$), and $\varepsilon$ is the default penalty associated with an individual unpaired species.

Finally, we also need to specify the default contribution to the cost function associated with species that are not paired (characterized by $\varepsilon$ in Eq. (2) and (3) of the main text). Since the algorithm used here allows species from either network to remain unpaired, this contribution defines an important threshold at which we permit non-pairing. Although the value of $\varepsilon$ is somewhat arbitrary, it needs to draw a "middle point" between equivalent and opposed motif-role profiles (see Eq. (1) in the Methods), penalizing unpaired species in order to incentivize the alignment between networks but avoiding the pairing of uncorrelated species. We set the contribution of unpaired species to $\varepsilon = 0$ because this represents null correlation according to the measure of pairwise role similarity used here.

## Alignment quality measures

In order for the alignments to be comparable across of networks, we also needed a size-independent measure of how good such alignment is. Finding this measure is not trivial since some motifs might only appear in big networks, artificially decreasing the overall similarity between what could be similar structures. Here we proposed four different measures of alignment quality:

- **Measure A.** First, we considered the measure described by Eq. (4) in the Methods section of the main text, which is a normalized version of Eq. (2).

- **Measure B.** The second measure that we define is a normalized version of Eq. (3) from the main text. Given the best alignment $\hat{\lambda}$ between webs $A$ and $B$, the quality of the alignment could be characterized by

$$\hat{e}_{AB}(\hat{\lambda}) = \frac{1}{N}\sum_{x\in\hat{\lambda}}\frac{1}{M_x}\left(\sum_{(\alpha,\beta)\in\hat{\lambda}_x}\big(1-\rho\left(\alpha,\beta\right)\big)+\min\left(k_{x_\alpha},k_{x_\beta}\right)\right), \quad (1)$$

where $\rho(a,\emptyset)=\rho(\emptyset,b)=1$, $M_x$ is the number of neighbors of species $a$ paired with neighbors of species $b$, and $N$ is the total number of matches between one species from $A$ and one species from $B$. Notice that this alignment quality normalizes all species' neighbors pairings while ignoring species that have not been paired.

- **Measure C.** We also consider a different measure of alignment quality that is not based on the ecological role of the species and does not use the pairwise similarity defined above. This is the proportional link overlap between aligned networks. Given an alignment between two food webs, the link overlap percentage tells us what percentage of links in one web are also present in the other one. Notice that if two networks $A$ and $B$ have a different number of links, an alignment between them can present two measures of the link overlap: the overlap of $A$ relative to $B$ and the overlap of $B$ relative to $A$. For example, given the alignment presented in Supplementary Fig. 10, the red network presents a 100% link overlap relative to the blue network and the blue networks presents a 75% link overlap with the red one. Since we are interested in a size independent measure of alignment quality, we focus here on the link overlap of the small network relative to the big one.

- **Measure D.** Given the alignment between two networks $A$ and $B$, the last measure is based around the idea of removing species for which the alignment algorithm could not find them a proper match. That is, we reduced the aligned networks so that only paired species were considered, and then we recalculated measure $A$. Doing this, we decrease the effect of the existence of species with different motif-role profiles only due to the size of the networks. For example, following the alignment presented in Supplementary Fig. 10, species $e$ would be removed and the resultant networks would present a perfect alignment. Notice that this measure differs from the first measure

because we now remove the motif formed by species $e$, $d$ and $c$ from the analysis; therefore, the post-removal motif-role profiles of every species-species pairing now perfectly match.

## Algorithm tests

*Effect of link and size differences*

We performed two tests for the alignment algorithm and alignment quality measures used in this study. In particular, we studied the effect of link and size differences on a random subset of networks. This is important because we need to check that the alignment quality measures are sensitive to changes in the structure of the networks but neutralize the effect of size difference between the aligned networks. First, we progressively randomized each of the selected networks—shifting them from the empirical networks to random graphs—and aligned the resultant randomized networks to their original versions (Supplementary Fig. 12). The alignment of a network with itself should present a perfect match; therefore, the alignment of a network with a randomized version of itself should highlight the differences in the way links are distributed within networks. Second, we performed a similar test to analyze how size differences alter the alignment between networks. To do so, we randomly removed a fraction of the species of each network and aligned the resultant networks to their original versions (Supplementary Fig. 12). In this case, the alignment of a network with one of its substructures should only highlight potential differences due to network size.

There are two crucial aspects of the tests that are important to analyze. First, the alignment quality measures should converge to a similar value when comparing the different empirical networks to random networks. This seems to be true in all the cases except for measure $C$, which clearly scales the network dissimilarity value with network size. This is not necessarily a problem for comparing differently sized networks; however, it will certainly add noise to the overall results. The second aspect to consider is the effect of size difference between networks when comparing identical structures. Ideally, the alignment quality measures should be able to neutralize this effect. We notice that measures $C$ and $D$ do a much better job neutralizing such effect than measures $A$ and $B$. Nevertheless, we also notice that the effect of size is specially relevant when removing random species from

small networks. The reason is because it is easier to break those networks into isolated links when randomly removing species. When focusing on the biggest networks of the test, one can notice that the alignment quality measures actually do a much better job neutralizing the effect of size.

*Alignment variability*

Another important aspect to assess regarding the alignment algorithm is the number of possible optimal alignments between two given networks. That is, the amount of variability found across alignments (see example in Supplementary Fig. 10). This is important because we want to know whether or not we need to align the networks multiple times in order to validate our results. Here, we aligned every pair of the networks used in the previous section 100 times and estimated the typical number of pairings of all species. For any given network, we found that the average number of pairings of a species is $2.03 \pm 1.88$, indicating that aligning networks only once is a reasonable approximation (Supplementary Fig. 10). In contrast, we found that this number is $20.52 \pm 10.82$ when aligning networks at random. As expected, however, we also found that the number of pairings increases when aligning bigger networks (Supplementary Fig. 10). Intuitively, this emerges since larger networks have a greater probability of including trophically identical species as well as species that participate in just a single interaction.

## Supplementary References


[1] Marti J Anderson. A new method for non-parametric multivariate analysis of variance. *Austral Ecol*, 26(1):32–46, 2001.

[2] Daniel B Stouffer and Jordi Bascompte. Compartmentalization increases food-web persistence. *Proc Natl Acad Sci USA*, 108(9):3648–3652, 2011.

[3] Elizabeth A Leicht and Mark EJ Newman. Community structure in directed networks. *Physical review letters*, 100(11):118703, 2008.

[4] Daniel B Stouffer, Marta Sales-Pardo, M Irmak Sirer, and Jordi Bascompte. Evolutionary conservation of species' roles in food webs. *Science*, 335(6075):1489–1492, 2012.

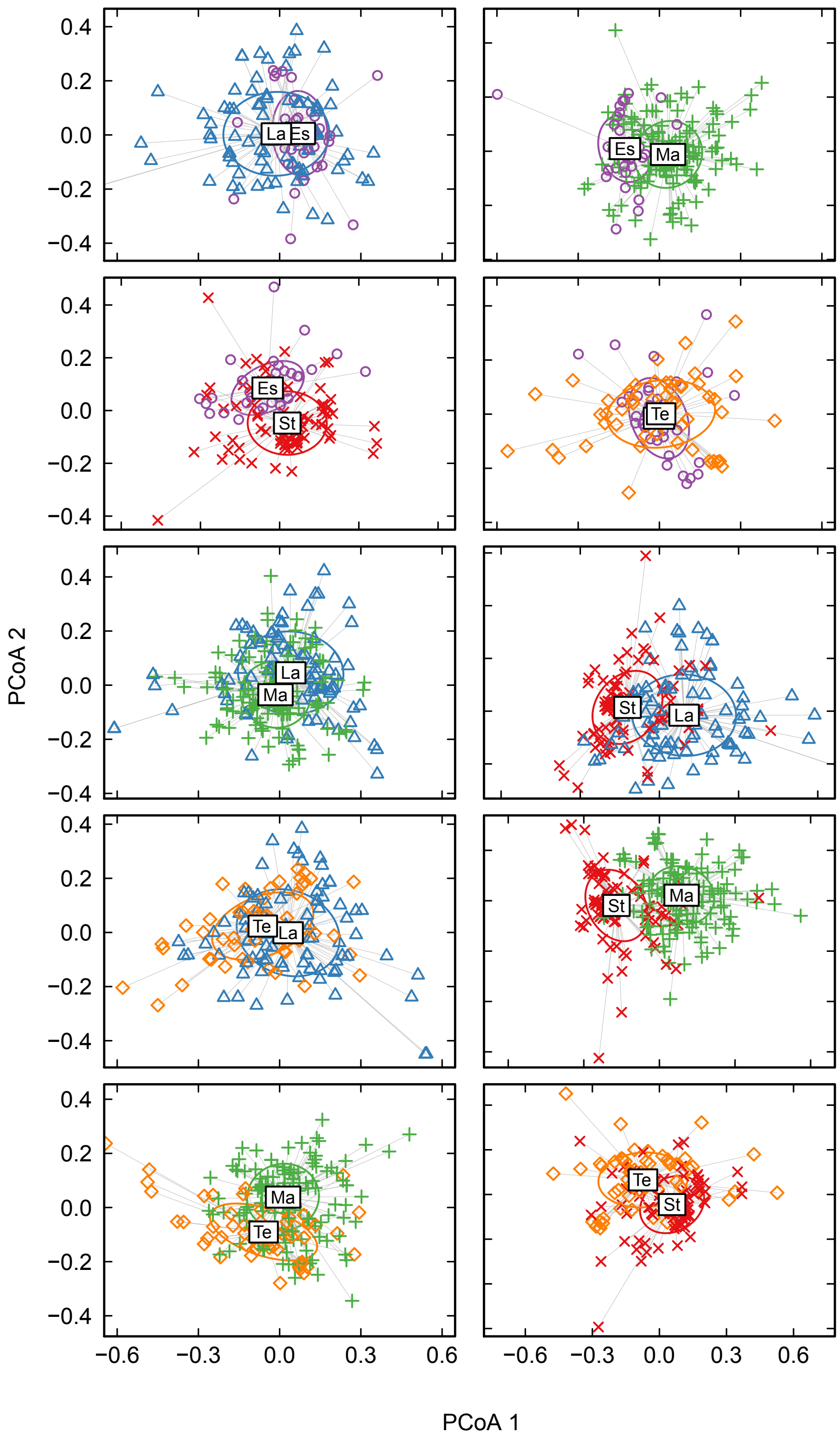


**Supplementary Figure 1**: Principal coordinate analysis for every pair of ecosystems. We used the alignment quality measure $A$, which is the measure used in the paper. As in Supplementary Fig. 2 of the main text, each different color and symbol characterizes the group of networks from estuaries (Es), lakes (La), marine (Ma), streams (St) and terrestrial (Te) ecosystems. The ellipses represent the 1 standard deviation ellipses about the group medians.

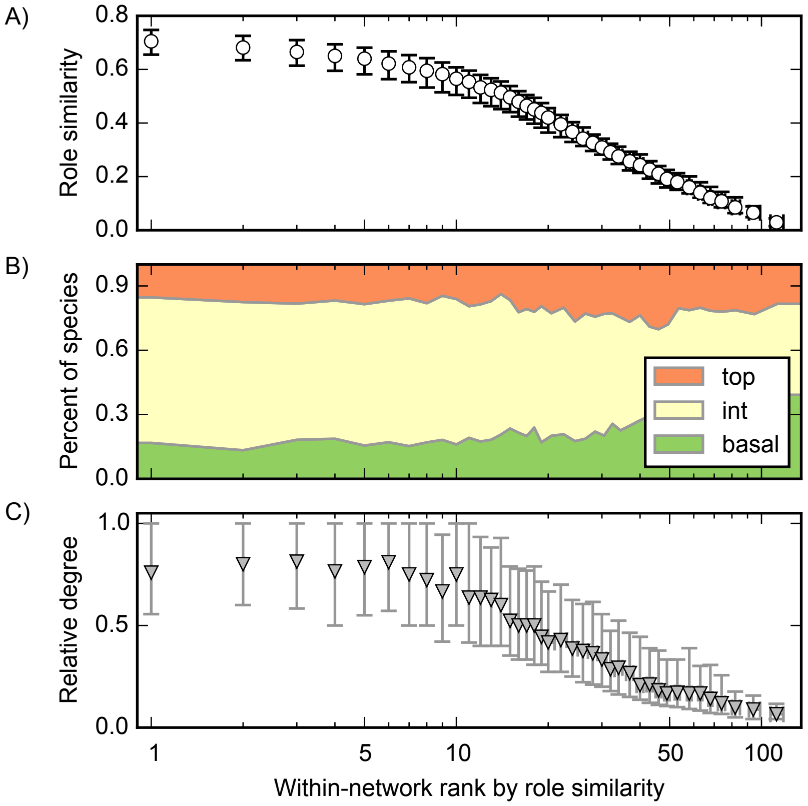


**Supplementary Figure 2**: Ranking of species from our dataset of 411 food webs based on the average similarity between their role and the roles of the species to which they are paired across all 84255 alignments. The top panel (A) shows the observed role similarity for all the species. The middle panel (B) describes the proportion of times that the ranked species are at top (i.e. not feeding on any species in the network), basal (i.e. not being consumed by any species in the network), and intermediate (i.e. feeding on and being consumed by other species in the web) trophic levels. The bottom panel (C) shows the relative degree of the ranked species. In all panels, every point indicates the median across at least 250 species with the exception of the last point which is the median across 30 species, and the bars characterize the inter quartile range.

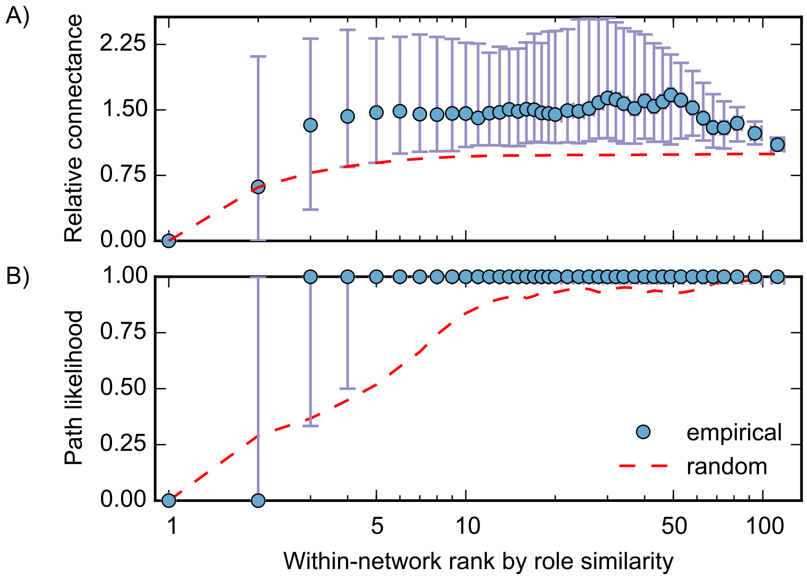


**Supplementary Figure 3**: Analysis of the connectance and path probability of the best aligned species of each network. The ranking of species is the same as the one presented in Supplementary Fig. 2. For every value of $x$, the blue circles represent (A) the relative connectance (connectance relative to the original network) and (B) path probability of the $x$ best aligned species of the ranking. The red dotted lines characterize the relative connectance and path probability expected for a random subset of $x$ species. In both panels, every point indicates the median across at least 250 species with the exception of the last point which is the median across 30 species, and the bars characterize the inter quartile range.

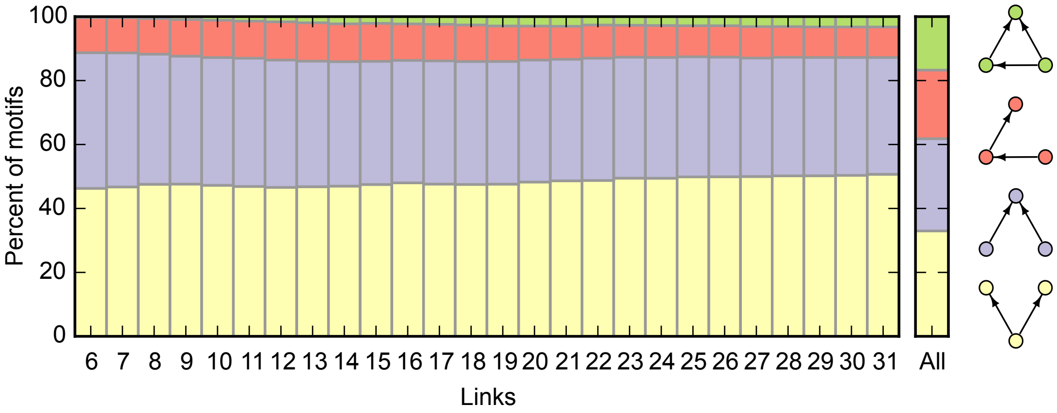


**Supplementary Figure 4**: Motifs analysis of the backbones of interactions. Each bar of the left plot describes the motif representation of the different $k$-link backbones of interactions found for every network in our dataset. The bar in the right plot describes the motif representation of the entire networks. The different colors characterize the proportion of each of the motifs found, which are represented on the right side of the figure. Notice that for the study of the motif structure of the entire networks, other motifs were found but in very low proportions, which make them imperceptible.

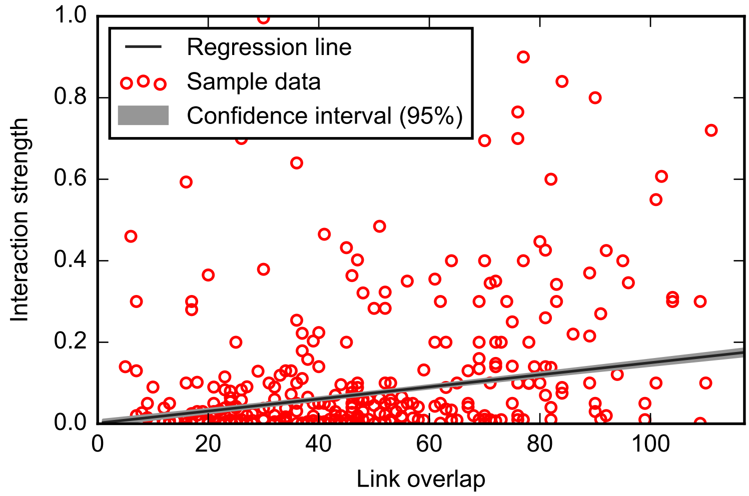


**Supplementary Figure 5**: Relationship between interaction strength and link overlap. The red dots are a random sample of the all the data. The black line characterizes the result a linear regression of the data ($F_{1,16610} = 818.1$, $p < 0.01$) and the gray area shows the corresponding 95% confidence intervals.

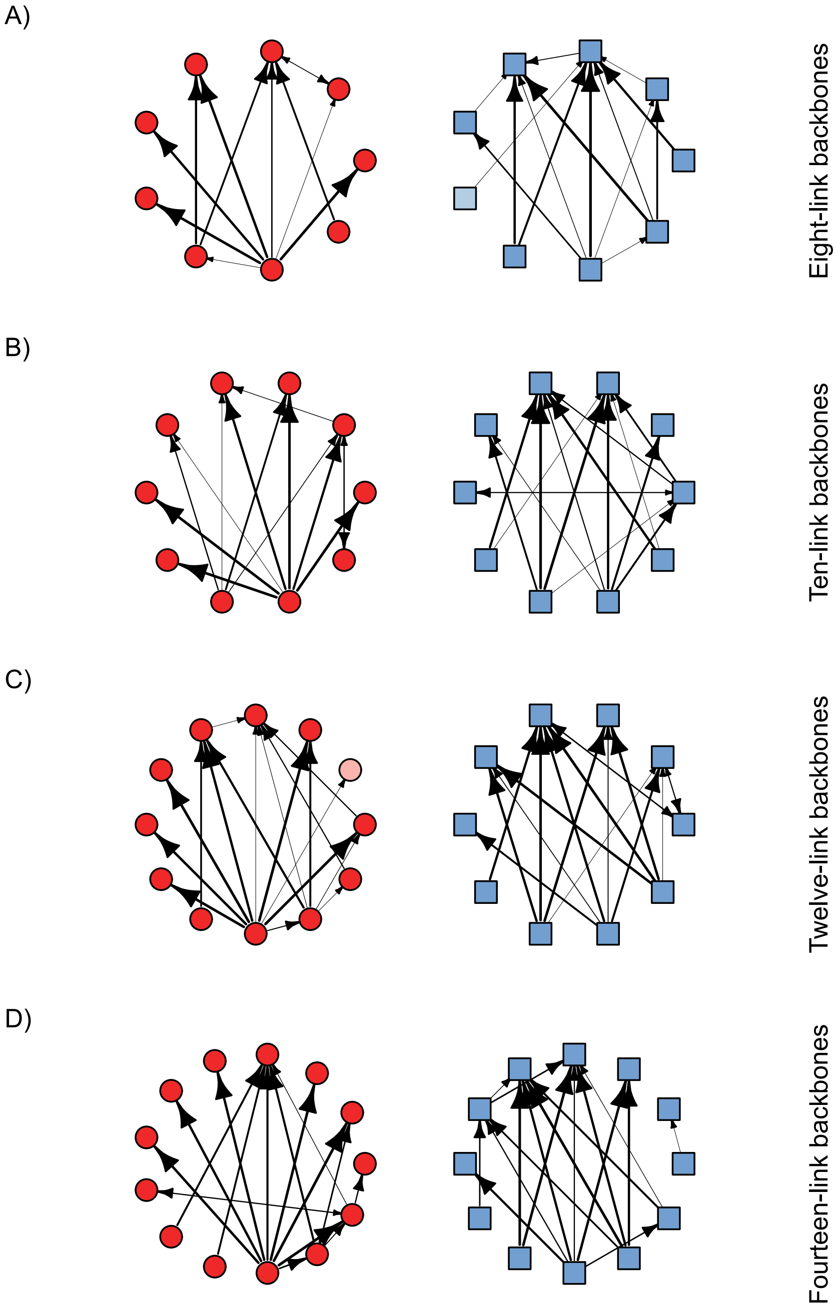


**Supplementary Figure 6**: Visualization of different-sized $k$-link backbones of interactions found across all food webs: (A) structures characterizing the 8-link backbones of interactions; (B) structures characterizing the 10-link backbones of interactions; (C) structures characterizing the 12-link backbones of interactions; and (D) structures characterizing the 14-link backbones of interactions. These structures are found by selecting the medoids of the clusters and overlapping them with all the within-cluster backbones, following the example shown in Fig. 1$C$ of the main text (see also Fig. 4 from the main text). The weight of the links is proportional to the likelihood $l$ of finding them in the backbones, and the light-shaded nodes represent nodes that significantly appear in the $k$-link backbones but not in the medoids. Note that links that were not significantly represented in the backbones ($l < 0.01$) are not shown.

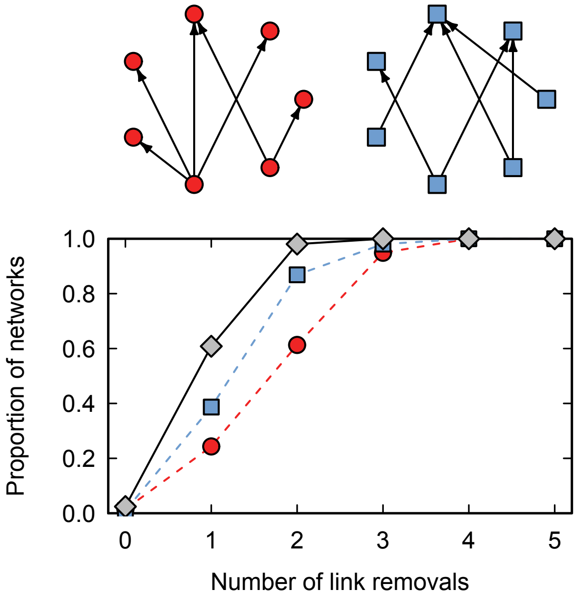


**Supplementary Figure 7**: Visualization of the backbones of interaction shared across food webs. The two networks depicted in this plot are the structures that encompass the greatest variation across our food-web dataset and can explain over 60% of the backbones within one link removal (see Supplementary Note 1). The dashed lines show the proportion of networks explained by each of these structure individually whereas the gray diamonds show the proportion of networks that those structure can explain in combination.

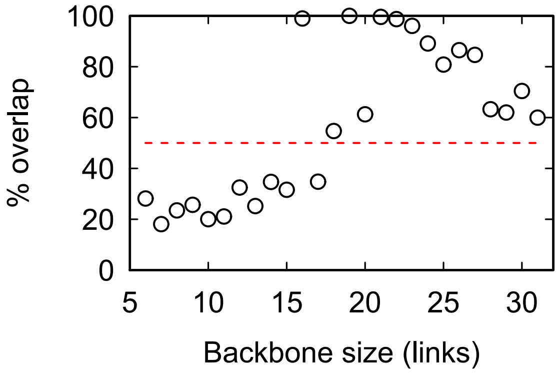


**Supplementary Figure 8**: Analysis of the quality of the backbone clustering. Every point represents the overlap of the 95% confidence ellipses that characterize the two clusters found for any given backbone size $k$. The red dotted line represents the 50% ellipse overlap.

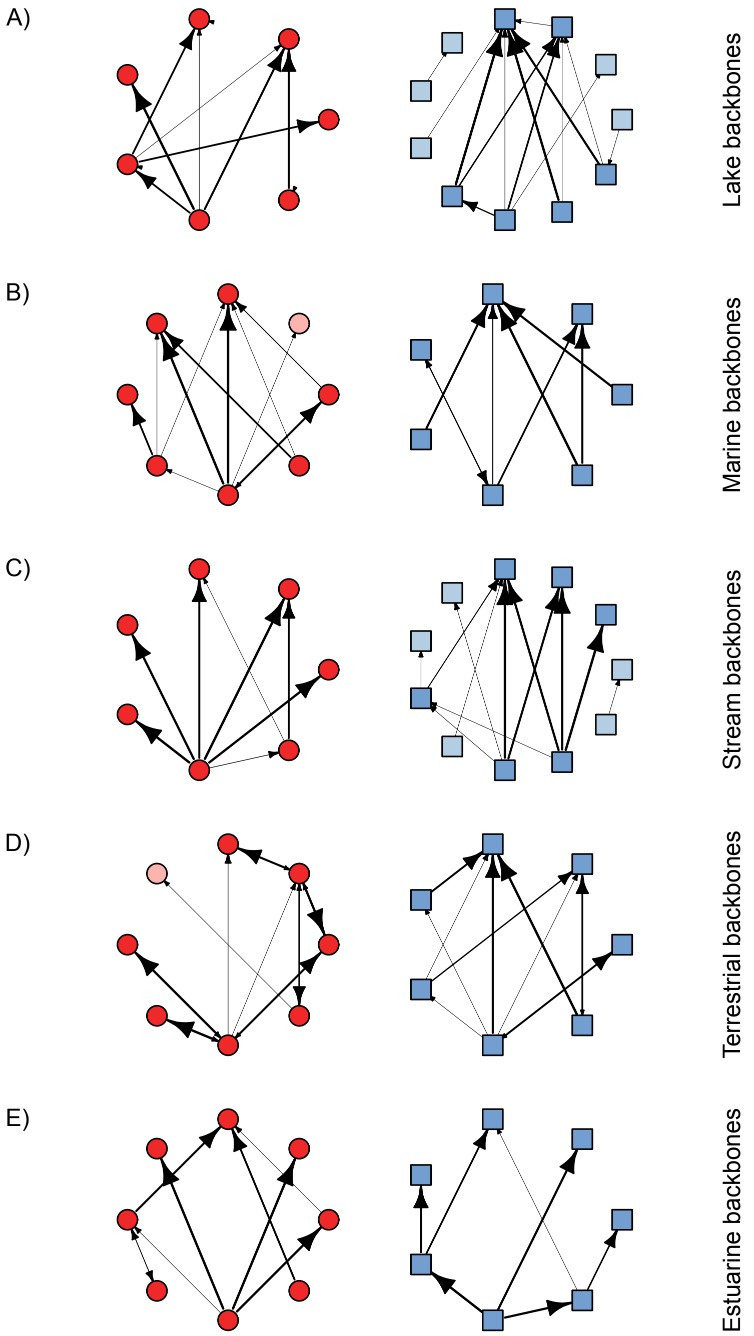


**Supplementary Figure 9**: Visualization of the 6-link backbones of interactions found across food webs from the different ecosystem types: (A) lake backbones; (B) marine backbones; (C) stream backbones; (D) terrestrial backbones; and (E) estuarine backbones. These structures are found by selecting the medoids of the clusters and overlapping them with all the within-cluster backbones, following the example shown in Fig. 1*C* of the main text (see also Fig. 4 from the main text). The weight of the links is proportional to the likelihood $l$ of finding them in the backbones, and the light-shaded nodes represent nodes that significantly appear in the $k$-link backbones but not in the medoids. Note that links that were not significantly represented in the backbones ($l < 0.01$) are not shown.

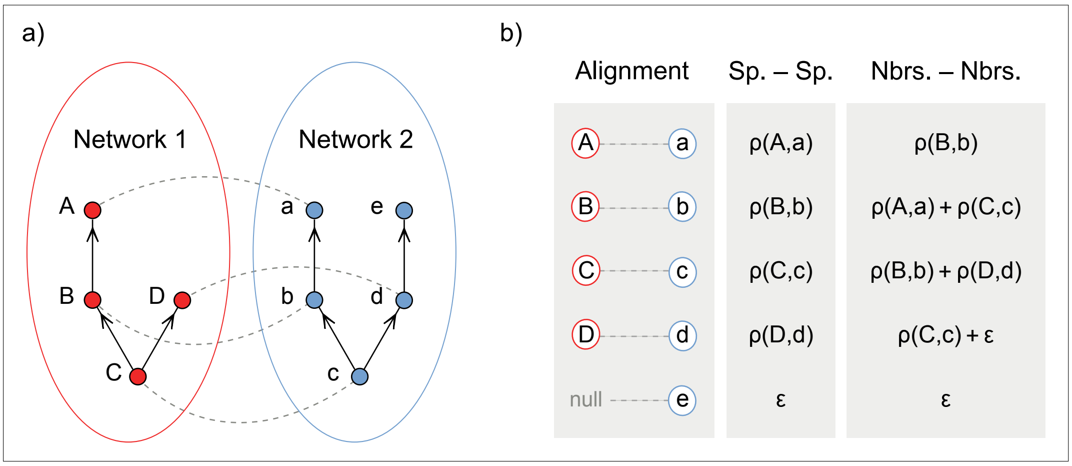


**Supplementary Figure 10**: Example of the pairwise role distance computation when pairing species to species and species' neighbors to species' neighbors. (a) Example of an alignment between two simple networks. Notice that in this example we consider one of the best possible alignments between the example networks. (b) A description of the pairwise distance between paired species according to both a species-species pairing and a neighbors-neighbors pairing. In this figure, $\rho(i, j)$ represents the role distance between species $i$ and $j$, and $\epsilon$ characterizes the default contribution of a unpaired species.

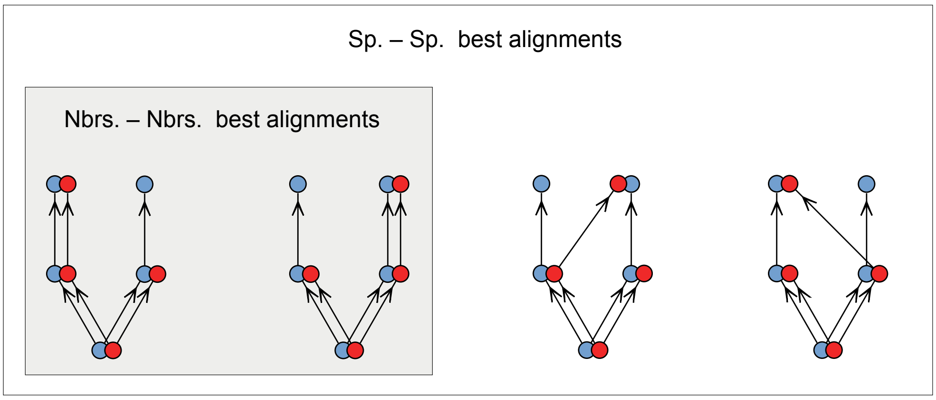


**Supplementary Figure 11**: Best possible alignments between networks 1 and 2 from Supplementary Fig. 10. Pairing species to species can be done by optimizing Eq. (2) of the main text, resulting into 4 indistinguishable alignments. Pairing species' neighbors to species' neighbors requires optimizing Eq. (2) of the main text and only produce 2 indistinguishable alignments.

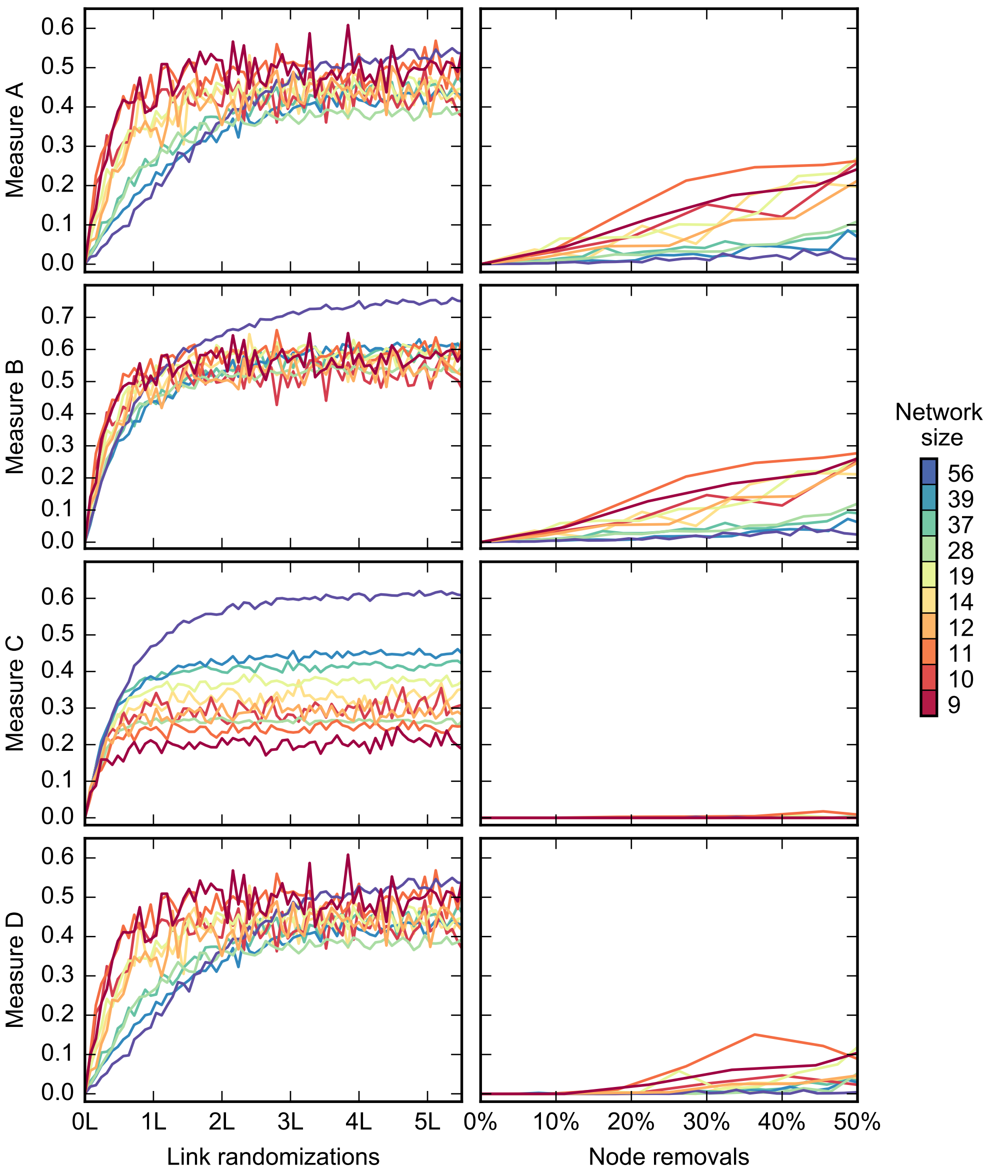


**Supplementary Figure 12**: Tests for the alignment algorithm and alignment quality measures presented in the previous section. The left panels show the results obtained when aligning the networks to random versions of themselves to test the effect of link differences between aligned networks. The number of link randomizations is expressed as a multiple of the total number of links $L$ of each network. The right panels show the results obtained when comparing networks to reduced versions of themselves to test the effect of size difference between aligned networks. The colors of the different lines are chosen based on the size of the networks.

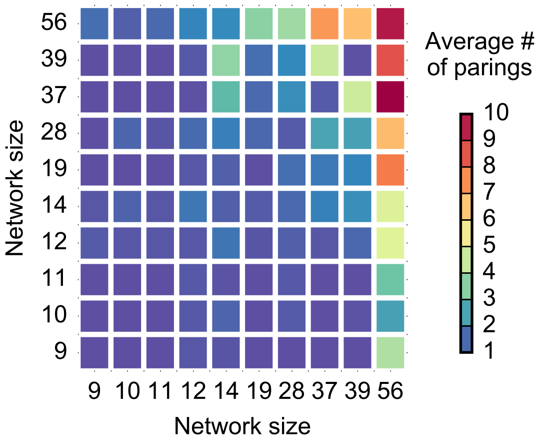


**Supplementary Figure 13**: Analysis of the alignment variability across networks. The panel shows the results obtained when optimally aligning 100 times every pair of networks used in the previous section. The color characterizes the average number of parings of any species of a given network. The networks are sorted based on their size (i.e. number of species).

| | | Measures of alignment quality | | | |
|---|---|---|---|---|---|
| | | A | B | C | D |
| PERMANOVA | Overall | 22.81 | 24.27 | 27.20 | 24.99 |
| | Size-constrained | 5.01 | 3.49 | -1.06 | 4.87 |

**Supplementary Table 1**: F-test statistic obtained using the PERMANOVA test when comparing the alignments between networks from 5 different ecosystems. In the overall analysis we compare 411 networks whereas in the size-constrained analysis we compare only 116 networks. The p-values found for each of these comparisons were $p < 0.01$ with the exception of the size-constrained statistical test performed on the size-constrained dissimilarity matrix (red value).

| | Estuarine | Lake | Marine | Stream | Terrestrial |
|---|---|---|---|---|---|
| Estuarine | | 4.24 | 18.43 | 14.93 | 0.76 |
| Lake | | | 6.19 | 36.42 | 8.01 |
| Marine | | | | 91.22 | 18.19 |
| Stream | | | | | 18.30 |

**Supplementary Table 2**: F-test statistic obtained using the PERMANOVA test when comparing network alignments between networks from different pairs of ecosystem types. In this case, we used the alignment quality measure $A$ to estimate the dissimilarity matrix. The p-values found for each of these comparisons were $p < 0.01$ with the exception of the comparison between estuarine and terrestrial food webs that was not statistically significant (red value).
